# The Chemokine CXCL7 Is Related to Angiogenesis and Associated With Poor Prognosis in Colorectal Cancer Patients

**DOI:** 10.3389/fonc.2021.754221

**Published:** 2021-10-08

**Authors:** Longhai Li, Kai Jiang, Dongpeng Li, Dongxiao Li, Zitong Fan, Guosheng Dai, Sheng Tu, Xiangyu Liu, Guangyou Wei

**Affiliations:** ^1^ Department of Science and Education, Bozhou Hospital of Anhui Medical University, Bozhou, China; ^2^ Department of Cardiovascular Medicine, Bozhou Hospital of Anhui Medical University, Bozhou, China; ^3^ Department of Pathology, Bozhou Hospital of Anhui Medical University, Bozhou, China; ^4^ Department of medicine, Anhui University of Science and Technology, Huainan, China; ^5^ Department of Otorhinolaryngology, Bozhou Hospital of Anhui Medical University, Bozhou, China

**Keywords:** CXCL7, VEGF, angiogenesis, prognosis, colorectal cancer

## Abstract

**Objective:**

The present study was designed to investigate the role of the chemokine CXCL7 in angiogenesis and explore its prognostic value in colorectal cancer (CRC).

**Methods:**

A total of 160 CRC patients who had undergone surgery were included in this study, and staged according to the guidelines of the AJCC, 7^th^ Edition. Expression of CXCL7 and VEGF was detected by immunohistochemical (IHC) staining and divided into high and low expression subgroups. The correlation between CXCL7 and VEGF expression was evaluated by Spearman’s rank-correlation coefficient. Prognosis based on CXCL7 and VEGF was evaluated using the Cox proportional hazards regression model and a nomogram of 5-year overall survival (OS) time.

**Results:**

CXCL7 was highly expressed in tumor tissues (65.63% *vs* 25.00% in paracancerous tissue, *P* < 0.001), as was VEGF. CXCL7 and VEGF expression correlated well with N and TNM stage cancers (all *P* < 0.001). Importantly, CXCL7 was positively correlated with VEGF expression in CRC tissues. CXCL7 was an independent predictor of poor OS of CRC patients (HR = 2.216, 95% CI: 1.069-4.593, *P* = 0.032), and co-expression of CXCL7 and VEGF of predicted poor OS of 56.96 months.

**Conclusion:**

Expression of CXCL7 correlated with VEGF and was associated with poor clinical outcomes in CRC patients.

## Introduction

Colorectal cancer (CRC) is one of the deadliest malignant tumors with over 1.8 million new cases worldwide each year, and contributes to about 10% of tumor-related deaths (1). In fact, 746,000 men and 614,000 women were diagnosed with CRC in developed countries, accounting for 55% of all cancer cases (2). Although the incidence of CRC has been stable and has actually declined in some developed countries owing to the improvements in lifestyle, environmental factors, medical and health conditions (3), new cases of CRC are expected to increase to 2.2 million and deaths to 1.1 million globally by 2030 (4). Therefore, it is vital to control the occurrence and development of CRC to better manage the substantial health burden. The incidence and mortality of CRC are increasing in China due to the aging population and advanced stages of the disease (5–7). Patients aged 75 years and over constitute a large proportion of new CRC cases (above 40%), and about 45% patients are in advanced stages when first diagnosed (8, 9). The 5-year survival rate is 92% in stage I CRC patients, but drops to 10% when patients are in advanced stage IV (10). Although progress in CRC diagnosis and treatment, including traditional surgical resection, radiotherapy, chemotherapy, and newly targeted immunotherapy, has been achieved, the long-term prognosis, especially in terms of 5-year survival rate, has not improved significantly in recent decades (11).

Angiogenesis is a physiological process whereby new blood vessels are generated from existing vessels, and is regulated by a variety of signaling pathways that dynamically balance pro- and anti-angiogenic responses under normal physiological conditions (12). However, angiogenesis can become abnormally active in response to injuries, the menstrual cycle, and some metabolic diseases (13). Many studies have shown that angiogenesis plays an important role in cancer by providing oxygen, nutrients and other factors in the tumor microenvironment to stimulate tumor growth and accelerate disease progression (14). Numerous factors are involved in angiogenesis, being roughly divided into two types: proangiogenic and antiangiogenic. Vascular endothelial growth factor (VEGF) and its corresponding receptors play an extremely important role in tumor angiogenesis. VEGF is a secreted glycoprotein dimer that promotes angiogenesis by binding to its receptors (15). VEGF exerts its biological functions by activating tyrosine protein kinase. Meanwhile, VEGF can increase capillary permeability, which allows fibrinogen and other proteins to diffuse into the extracellular matrix where they crosslink into a fibrous gel, thus effectively promoting formation of a capillary network (15, 16). VEGF is highly expressed in many tumors where it is the strongest driver of angiogenesis, thus contributing to tumor growth and metastasis (17, 18).

Chemokines are low-molecular-weight signaling proteins (8–12 kDa) that can regulate many biological processes, including cellular immune response, glucose metabolism and angiogenesis (19–22). Chemokines play important regulatory roles by binding to their specific receptors (23). Recently, numerous studies have shown that various chemokines play important roles in tumorigenesis, including regulation of proliferation, tumor metastasis, angiogenesis, among others (24–27). Chemokines also affect cancer diagnosis and treatment by interacting with tumor cells and the tumor immune environment (28, 29). CXCL7, also called neutrophil activating peptide 2, functions by binding to its receptors CXCR1 and CXCR2, and plays important roles in a variety of tumor processes (30–32).

Our previous study showed that CXCL7 is highly expressed in CRC with poor prognosis (30, 33). However, the role of CXCL7 in angiogenesis is unknown. In the current study we probed the function of CXCL7 in angiogenesis and evaluated its prognostic value by analyzing clinical data from a total of 160 CRC patients, including 2 in Stage I, 67 in Stage II, 69 in Stage III and 22 in Stage IV CRC. We assessed the correlation between CXCL7 and VEGF expression and systematically explored the value of CXCL7 for prognosis in CRC.

## Materials and Methods

### Patient Selection and Tissue Sample Collection

The subjects were enrolled according to the following criteria. (1) Patients were firstly diagnosed in Bozhou Hospital of Anhui Medical University from July 2013 to July 2018. (2) Patients whose condition met the criteria for surgical resection were enrolled in the study. (3) Preoperative clinical diagnosis was consistent with postoperative pathological diagnosis in indicating colorectal cancer. (4) Patients had not received radiotherapy and chemotherapy prior to surgery. (5) The patient’s clinical data was relatively complete. Other terms and selection criteria for healthy participants were described in our previous study (30, 33). These criteria were met by 200 patients. However, 40 people were not eligible during the follow-up. Therefore, 160 patients were included in this study finally (**Supplementary Figure 1**). Patient data included demographic characteristics and clinical pathology results. All patients were staged according to AJCC (American Joint Committee on Cancer) guidelines, 7^th^ Edition (34). The study was approved by the Institutional Research Ethics Committee of our hospital, and 160 written informed consents were obtained. Tumor and adjacent non-tumor tissues were collected from all 160 patients, then fixed by formalin and embedded in paraffin.

### Immunohistochemical Staining Assay

Serial sections of 4 μm thickness were cut from each of the 320 paraffin blocks. IHC staining was performed according to the manufacturer’s instructions using anti-CXCL7 mouse monoclonal antibody (1:200, Abcom Ltd., Cam-bridge, UK) and anti-VEGF rabbit monoclonal antibody (1:300, Abcom Ltd., Cam-bridge, UK). The IHC process made use of an automated immunostainer (Thermo Fisher Scientific, MA, USA). IHC staining was scored by two pathologists, and scores were only accepted when they were in agreement. Light microscopy images were obtained from each slice at 40× magnification. Overall IHC staining was scored as either 0 (none), 1 (weak), 2 (moderate), or 3 (strong). The percentage of positively-stained cells was recorded as either 0 (≤5%), 1(6%–25%), 2(26%–75%), or 3(>75%). The final score was 0–9, calculated as the product of the scores for overall IHC staining and percentage positive staining. The cutoff value for IHC staining was calculated using the receiver operating characteristic (ROC) curve and the Youden Index (sensitivity+specificity-1). The final score was taken to represent high expression when it was greater than the cutoff value, and low expression when less than or equal to the cutoff.

### Methods of Postoperative Follow-Up and Collection of Related Information

Two doctors were responsible for follow-up and collection of patient information. Postoperative survival times of CRC patients were collected at 3 months and 1, 2, 3, 5, and 8 years after surgery; follow-up was halted if the patient died. Patient data was categorized according to (1) CXCL7 and VEGF staining in cancer and adjacent normal tissues, (2) age and gender, and (3) pathological parameters (30).

### Statistical Analysis

The results of categorical variables were presented as frequencies (n) and percentages (%) in this study. The mean (M) and standard deviation (SD) were used to represent numerical variables. Expression of CXCL7 and VEGF were sequentially tested in different variables by Pearson’s chi-square test. Differences between CXCL7 and VEGF expression were analyzed by the Mann-Whitney U-test. Spearman’s rank-correlation test was used to evaluate the correlation between CXCL7 and VEGF expression. The 5-year overall survival (OS) time was calculated using Kaplan-Meier analysis, and graphs of survival rate are shown in Results. Cox proportional hazard regression models were used for analyzing risk factors used in prognoses for CRC patients. First, statistically significant indicators were selected by univariate analysis. Then, multivariate regression models were employed to seek out independent prognostic factors. R 4.0.0 software was used to draw a nomogram of 5-year OS. In addition, the concordance index (C-index), the area under the ROC curve (AUC), and the calibration curve were used to evaluate the predictive ability of the nomogram model. SPSS 22.0 software (IBM; Armonk, NY, USA) was used to analyze all data in this study. Statistical differences were significant for *P* value < 0.05.

## Results

### General Clinical Information of CRC Patients

One hundred and sixty patients who had been definitively diagnosed with CRC were recruited, including 73 women (45.63%, 73/160) and 87 men (54.37%, 87/160). The average age was 59.46 ± 9.13 years for women and 58.17 ± 11.24 years for men (**Table 1**). Patients were classified into two groups based on age: ≤ 60 (55.00%, 88/160) and > 60 (45.00%, 72/160). Other CRC patient information was shown in **Table 1**.

**Table 1:** Association of CXCL7 and VEGF with clinical pathological characteristics and the correlation between two markers in CRC patients.

Clinicopathologic parameters	Case (n = 160)	CXCL7 expression		*P*-value	VEGF expression		*P*-value	r	*P*-value
		Low	High		Low	High			
Total	160	55	105		57	103		0.796	**<0.001**
Gender				0.975			0.739		
Male	87	30	57		25	48		0.760	**<0.001**
Female	73	25	48		32	55		0.842	**<0.001**
Age				0.452			0.584		
≤60	88	28	60		33	55		0.815	**<0.001**
>60	72	27	45		24	48		0.765	**<0.001**
Tumor location				0.532			0.525		
Colon	76	28	48		29	47		0.815	**<0.001**
Rectum	84	27	57		28	56		0.774	**<0.001**
Cancer site				0.696			0.070		
Left	125	42	83		40	85		0.788	**<0.001**
Right	35	13	22		17	18		0.763	**<0.001**
Tumor size				0.304			0.308		
<4cm	70	21	49		28	42		0.817	**<0.001**
≥ 4cm	90	34	56		29	61		0.775	**<0.001**
Depth of tumor invasion				0.133			0.204		
T1-T2	49	21	28		21	28		0.765	**<0.001**
T3-T4	111	34	77		36	75		0.823	**<0.001**
Lymph node metastasis				**<0.001**			**<0.001**		
N0	76	39	37		43	33		0.726	**<0.001**
N1-N2	84	16	68		14	70		0.586	**<0.001**
Distant metastasis				0.366			0.574		
M0	137	49	88		50	87		0.778	**<0.001**
M1	23	6	17		7	16		0.523	**<0.001**
TNM stage				**<0.001**			**<0.001**		
I-II	69	36	33		39	30		0.693	**<0.001**
III-IV	91	19	72		18	73		0.609	**<0.001**
Neural invasion				0.504			0.212		
No	105	38	67		41	64		0.688	**<0.001**
Yes	55	17	38		16	39		0.829	**<0.001**
Vascular invasion				0.658			0.302		
No	59	19	40		18	41		0.675	**<0.001**
Yes	101	36	65		39	62		0.848	**<0.001**
Differentiation				0.625			0.636		
Well	77	25	52		26	51		0.787	**<0.001**
Moderate-Poor	83	30	53		31	52		0.795	**<0.001**

Bold values mean that the value is statistically significant (P < 0.05).

### Expression of CXCL7 and VEGF in CRC Tissues

**Figure 1:**
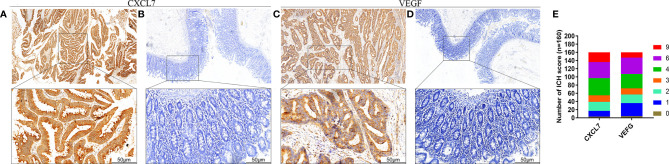
Expression of CXCL7 and VEGF in CRC patients. **(A)** Expression of CXCL7 in tumor tissues (magnification: 40× and 200×). **(B)** Expression of CXCL7 in normal tissues (40× and 200×). **(C)** Expression of VEGF in tumor tissues (40× and 200×). **(D)** Expression of VEGF in normal tissues (40× and 200×). **(E)** Patients were classified according to CXCL7 and VEGF IHC score from 0 to 9. Scale bar, 50 μm.

IHC was used to monitor expression of CXCL7 and VEGF in CRC tumors and adjacent normal tissues. Based on the ROC curve, a score of 3.5 was selected as the cutoff value for CXCL7, and 2.8 for VEGF. Similar to our previous research, CXCL7 was highly expressed in tumor tissues (65.63%, 105/160) but not in paracancerous tissue (25.00%, 40/160, *P* < 0.001; **Figures 1A, B**). Positive staining was mainly concentrated in the cytoplasm and membranes of CRC cells (**Figure 1A**). Similarly, we found that VEGF was also highly expressed in CRC tissues (64.38%, 103/160 *vs*. 26.25%, 42/160 in normal tissues, **Figures 1C, D**). Number of ICH score was shown in **Figure 1E**.

### Associations Between CXCL7, VEGF and Clinicopathological Characteristics in CRC Patients

**Figure 2:**
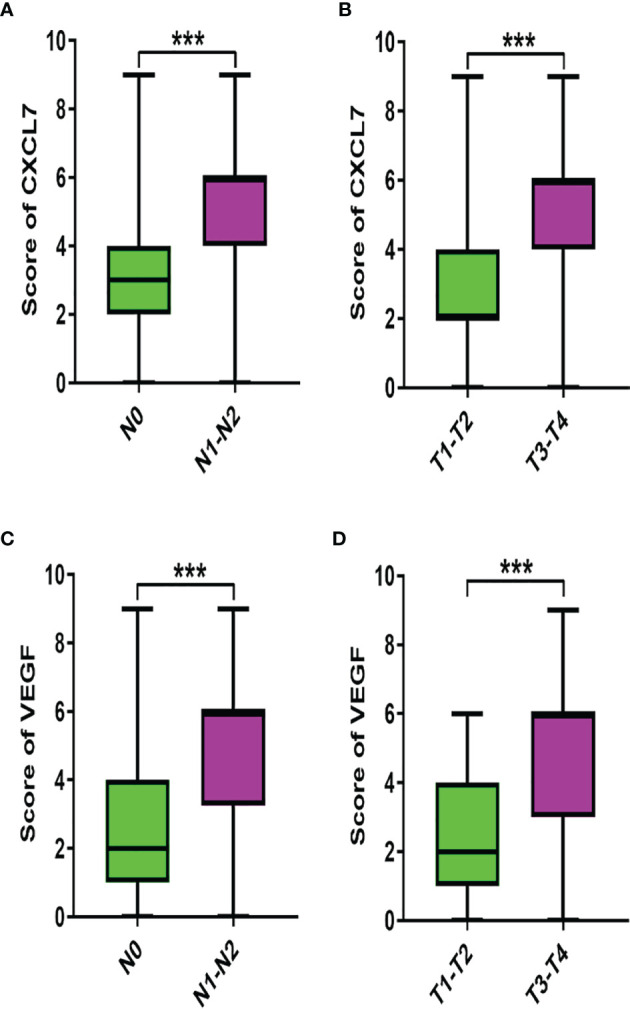
Associations between CXCL7 and VEGF and clinicopathological characteristics. **(A)** Expression of CXCL7 in different N-stage tumors. **(B)** Expression of CXCL7 in different TNM stages. **(C)** VEGF in N0 and N1-N2 stage tumors. **(D)** VEGF in I-II and III-IV stages. The Mann‐Whitney U-test was used for analysis. ***P < 0.001.

In order to probe the clinical significance of CXCL7 and VEGF in CRC, associations between these two markers and clinicopathological information were analyzed by the chi-square (χ^2^) test, and differences in expression were assessed by the Mann-Whitney U test. CXCL7 was correlated with N- and TNM-stage cancer (both *P* < 0.001; **Figures 2A, B** and **Table 1**), as was VEGF (both *P* < 0.001; **Figures 2C, D** and **Table 1**). However, there were no significant correlations between CXCL7 or VEGF and other clinicopathological characteristics (all *P* > 0.05; **Table 1**).

### Clinical Correlation Between CXCL7 and VEGF in CRC Patients

**Figure 3:**
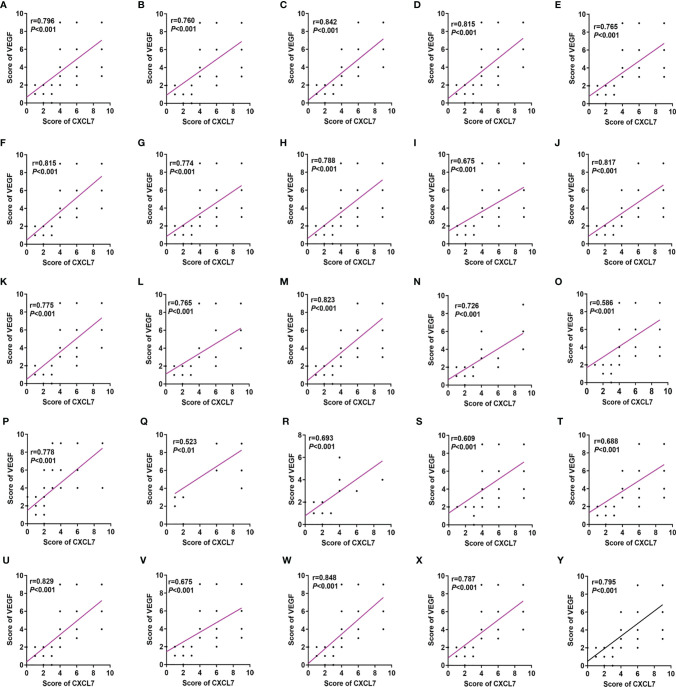
CXCL7 positively correlated with VEGF in CRC patients grouped according to various clinical features. **(A)** All CRC patients. **(B)** Male subgroup. **(C)** Female subgroup. **(D)** Age ≤ 60 years subgroup. **(E)** Age > 60 years subgroup. **(F)** Colon subgroup. **(G)** Rectum subgroup. **(H)** Left subgroup. **(I)** Right subgroup. **(J)** Tumor size <4 cm subgroup. **(K)** Tumor size ≥4 cm subgroup. **(L)** T1-T2 subgroup. **(M)** T3-T4 subgroup. **(N)** N0 subgroup. **(O)** N1-N2 subgroup. **(P)** M0 subgroup. **(Q)** M1 subgroup. **(R)** I-II subgroup. **(S)** III-IV subgroup. **(T)** Non-neural invasion subgroup. **(U)** Neural invasion subgroup. **(V)** Non-vascular invasion subgroup. **(W)** Vascular invasion subgroup. **(X)** Well differentiated subgroup. **(Y)** Moderately-poorly differentiated subgroup. The non-parametric Spearman’s test was used for these analyses.

To analyze whether expression of CXCL7 was related to the level of VEGF in CRC tissue, Spearman’s test was used to evaluate the correlation. Interestingly, there was a significant correlation between CXCL7 and VEGF in all CRC tissues (correlation coefficient: 0.796, *P* < 0.001, **Figure 3A**). We then explored whether there were correlations with other clinical features. Scatter plots were used to visualize results for the two markers. As shown in **Figure 3**, the correlation coefficients ranged from 0.523 to 0.842 (all *P* < 0.01), with the highest being for the female subgroup (r= 0.842) and the lowest for the M1 subgroup (r= 0.523, **Figures 3A–Y** and **Table 1**). These results illustrated a strong correlation between CXCL7 and VEGF in CRC tissues.

### High Expression of CXCL7 and VEGF Related to Poor Prognosis in CRC Patients

**Figure 4:**
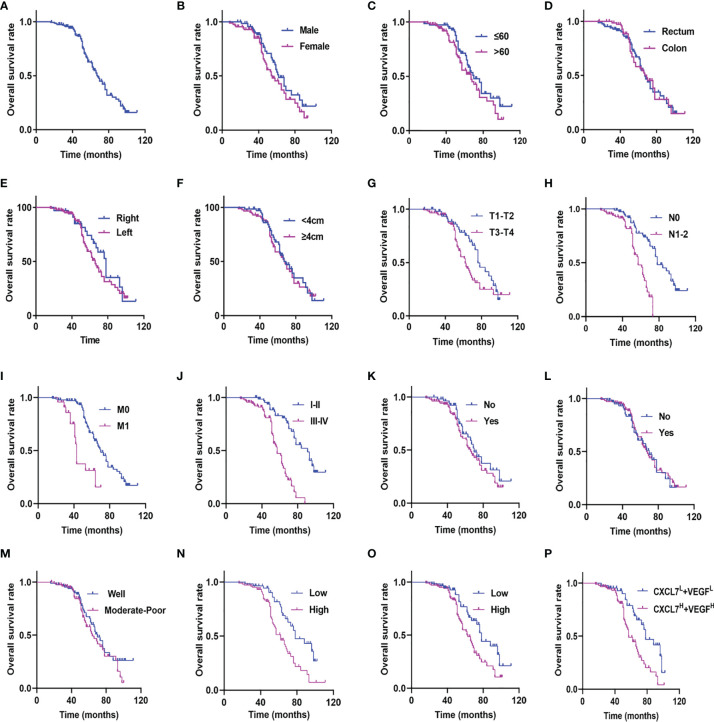
Kaplan-Meier curves for CRC patients stratified on the basis of CXCL7, VEGF and clinicopathological features. **(A)** OS in all 160 patients. **(B)** OS in male *vs*. female patients (*P* = 0.133). **(C)** OS in age ≤60 *vs*. age >60 patients (*P* = 0.117). **(D)** OS in colon *vs*. rectum patients (*P* = 0.899). **(E)** OS in left side *vs*. right side patients (*P* = 0.252). **(F)** OS in tumor size <4 cm *vs*. ≥4 cm patients (*P* = 0.740). **(G)** OS in T1-T2 *vs*. T3-T4 patients (*P* = 0.032). **(H)** OS in N0 *vs*. N1-N2 patients (*P* < 0.001). **(I)** OS in M0 *vs*. M1 patients (*P* < 0.001). **(J)** OS in I-II *vs*. III-IV patients (*P* < 0.001). **(K)** OS in non-neural invasion *vs*. neural invasion patients (*P* = 0.271). **(L)** OS in non-vascular invasion *vs*. vascular invasion patients (*P* = 0.859). **(M)** OS in well differentiated *vs*. moderately-poorly differentiated patients (*P* = 0.271). **(N)** OS in CXCL7 low expression (CXCL7^L^) *vs*. CXCL7 high expression (CXCL7^H^) patients (*P* = 0.001). **(O)** OS in VEGF low expression (VEGF^L^) *vs*. VEGF high expression (VEGF^H^) patients (*P* = 0.008). **(P)** OS in CXCL7^H^ + VEGF^H^ *vs*. CXCL7^L^ +VEGF^L^ patients (*P* = 0.001).

For the purpose of establishing the prognostic significance of CXCL7 and VEGF in CRC, the 160 patients were observed to have a median OS time of 67.00 (95% confidence interval [CI]: 61.37–72.63) months and a 5-year survival rate of 62.60% (**Figure 4A**). Interestingly, OS time was affected by extent of tumor invasion. There were significant differences in OS among the tumor stages: T (hazard ratio [HR]: 1.711; 95% CI: 1.048–2.793; *P* =0.032; **Figure 4G**), N (HR: 3.717; 95% CI: 2.160-6.397; *P <*0.001; **Figure 4H**), M (HR: 3.696; 95% CI: 1.991–6.862; *P <*0.001; **Figure 4I**), TNM (HR: 3.765; 95% CI: 1.828–7.756; *P <*0.001; **Figure 4J**), and level of CXCL7 (HR: 2.343; 95% CI: 1.431–3.837; *P* =0.001; **Figure 4N**) and VEGF (HR: 1.931; 95% CI: 1.189–3.136, *P* = 0.008; **Figure 4O**). Details are shown in **Table 2**. However, no significant differences were found among the other factors (**Figures 4B–F, K–M** and **Table 2**; all *P >*0.05). Multivariate analysis showed that high CXCL7 in tissues was independent of factors associated with poor OS of CRC patients (HR=2.363; 95% CI: 1.359–4.108; *P* = 0.002; **Table 2**). Patients with high levels of both CXCL7 and VEGF had poor outcomes (OS: 56.96 months *vs* 78.00 months in patients with low levels of each, HR: 2.410; 95% CI: 1.405-4.132; *P* = 0.001; **Figure 4P**).

**Table 2:** Cox proportional hazard regression models for CXCL7, VEGF and clinical pathological characteristics.

Clinicopathologic parameters		Median of OS (95% CI)	5-year OS (%)	Univariate analysis		Multivariate analysis	
				HR (95% CI)	*P*-value	HR (95% CI)	*P*-value
Total		67.00 (61.37-72.63)	62.60				
Gender	Male	62.08 (50.08-74.12)	55.70	1.406 (0.901-2.194)	0.133		
	Female	70.00 (61.82-78.18)	70.50				
Age	≤60	70.00 (57.48-82.53)	71.20	0.699 (0.448-1.093)	0.117		
	>60	67.00 (54.09-79.91)	55.40				
Tumor location	Colon	67.00 (56.85-77.15)	58.00	0.972 (0.626-1.509)	0.899		
	Rectum	67.00 (59.68-74.32)	67.10				
Cancer site	Left	66.00 (60.27-71.33)	59.20	0.733 (0.432-1.246)	0.252		
	Right	78.00 (69.97-86.03)	70.50				
Tumor size	<4 cm	67.00 (55.27-78.73)	65.50	1.078 (0.691-1.684)	0.740		
	≥ 4 cm	67.00 (60.27-73.73)	57.10				
Depth of tumor invasion	T1-T2	76.00 (66.38-85.63)	78.20	1.711 (1.048-2.793)	**0.032**	1.421 (1.043-1.936)	**0.026**
	T3-T4	62.18 (55.19-69.17)	55.10				
Lymph node metastasis	N0	78.00 (65.22-90.78)	75.40	3.717 (2.160-6.397)	**<0.001**	1.250 (0.828-1.886)	0.288
	N1-N2	56.92 (50.61-63.23)	47.40				
Distant metastasis	M0	70.00 (63.28-76.74)	66.80	3.696 (1.991-6.862)	**<0.001**	1.330 (0.549-3.219)	0.528
	M1	42.93 (41.17-44.70)	15.60				
TNM stage	I-II	84.00 (64.02-103.98)	74.10	3.765 (1.828-7.756)	**<0.001**	2.363 (1.359-4.108)	**0.002**
	III-IV	55.70 (51.47-59.93)	37.30				
Neural invasion	No	70.00 (62.26-77.47)	64.90	1.302 (0.812-2.095)	0.271		
	Yes	66.00 (58.57-73.43)	60.00				
Vascular invasion	No	70.00 (59.72-80.28)	62.30	0.959 (0.605-1.521)	0.859		
	Yes	67.00 (59.74-74.26)	60.60				
Differentiation	Well	70.00 (62.44-77.34)	67.50	1.304 (0.812-2.095)	0.271		
	Moderate-Poor	64.00 (56.01-71.99)	57.70				
CXCL7	Low	78.00 (61.77-94.83)	79.20	2.343 (1.431-3.837)	**0.001**	1.990 (1.162-3.406)	**0.012**
	High	62.00 (54.21-69.79)	49.30				
VEGF	Low	78.00 (72.53-83.48)	74.30	1.931 (1.189-3.136)	**0.008**	1.709 (0.984-2.969)	0.057
	High	62.18 (53.78-70.58)	53.10				
CXCL7 low +VEGF low		78.00 (62.34-93.66)	78.60	2.410 (1.405-4.132)	**0.001**		
CXCL7 high +VEGF high		56.96 (50.16-63.77)	46.40				

Bold values mean that the value is statistically significant (P < 0.05).

### A Prognostic Nomogram for Comprehensively Evaluating OS in CRC Patients

**Figure 5:**
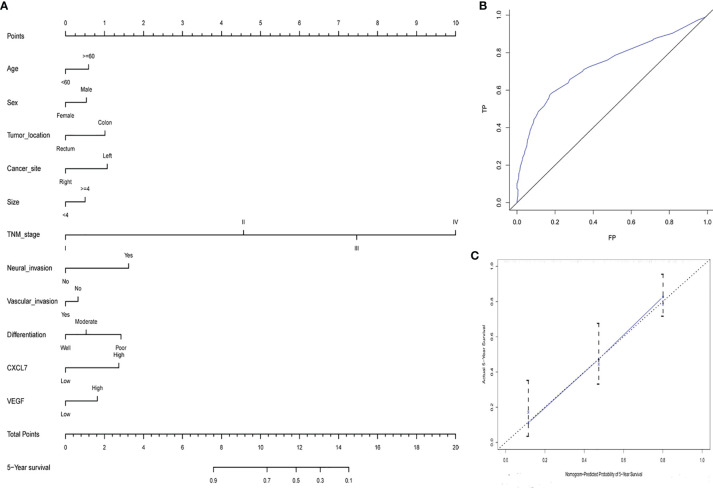
Nomogram of CRC patients’ 5-year survival time following surgery. **(A)** Nomogram for 5‐year OS on the basis of CXCL7 and VEGF expression and basic clinical data. **(B)** ROC curve for developing the nomogram. **(C)** Calibration curve of the nomogram.

As nomograms are widely used in prognostic assessments, especially for cancer, we generated a nomogram using the R software package to analyze the value of CXCL7, VEGF and various pathological features in making a CRC prognosis (**Figure 5A**). First, a standard point-scale reference line was established on the top of the nomogram. Then, CXCL7, VEGF and related clinical and pathological information were listed in order. The total point score was then calculated for prediction of 5-year OS (only 5-year OS was evaluated in this study). From the nomogram, patients could be provided with a personalized evaluation for guiding clinical treatment following surgery. The length of each variable was associated with patient survival. TNM stage, neural invasion, CXCL7 expression and differentiation had the greatest impact on prognosis.

The nomogram was also quantitatively assessed using the concordance index (C-index), area under the ROC curve (AUC), and calibration curve. The C-index was 0.747 (95% CI: 0.701–0.826). The 5‐year survival AUC was 0.734 (95% CI: 0.672–0.797; **Figure 5B**), and the calibration curve was shown in **Figure 5C**. Overall, the nomogram for 5-year OS could be used to make a reliable prognosis for CRC patients.

## Discussion

CRC is a serious public health challenge associated with high mortality and morbidity worldwide, especially in developing countries such as China (35). Although numerous improvements related to diagnosis and treatment have been applied in clinical practice, the number of tumor-related deaths has continued to rise over the past few years (36). Surgery and postoperative radiotherapy and chemotherapy are standard treatments for CRC. Improvement of OS following surgery for CRC requires systematic evaluation and management (37). TNM stage has been used as a predictor of survival time in recent decades (38), although it is not always accurate. For this reason, recurrence and metastasis after treatment are usually included as risk factors for prognosis. Of course, a great many studies in the past few decades have identified novel prognostic biomarkers (39–41). Various studies have found that angiogenesis plays an important role in recurrence and metastasis (42, 43). Even though many proteins and signaling molecules have been associated with angiogenesis in CRC cells (44, 45), the connection between CXCL7 and angiogenesis has not previously been explored. In this study, we performed series of experiments to evaluate the association of CXCL7 with angiogenesis, and assess its value in prognosis of CRC patients.

CXCL7, a neutrophil-activating chemokine, is primarily derived from peripheral platelets (46). Traditionally, CXCL7 has been considered to be involved in regulating glycolysis, mitosis, and prostaglandin synthesis, among other processes (46, 47). Recently, several studies have reported that CXCL7 is associated with many types of tumors, and plays important roles in tumor proliferation and metastasis (48, 49). We used IHC to show that CXCL7 was significantly over-expressed in CRC tumor cells, correlated with N-stage and TNM-stage cancer, and associated with poor patient outcomes. CXCL7 was evaluated as a CRC biomarker in our previous study (30), and together with the present study has been shown be a potentially reliable marker for making prognoses in CRC patients.

Angiogenesis, an important physiological process, also plays a pivotal role in tumor proliferation, invasion and metastasis (12, 13). A variety of proteins are involved in regulation of angiogenesis, including VEGF and other cytokines and chemokines (13, 14). Here, VEGF was highly expressed in CRC tumors and correlated with poor prognosis. As the role of VEGF in angiogenesis is widely known, anti-VEGF treatment drugs such as bevacizumab and many traditional plant polysaccharides (50, 51) are commonly used in anti-cancer treatments.

Previously we have focused on CXCL7 in serum from CRC patients (30), and its potential application to prognosis of obstructive colorectal cancer (33); however, the relationship between CXCL7 and angiogenesis has not previously been evaluated. In recent years, the role of chemokines in regulating angiogenesis has been revealed in several studies (52, 53), including the ability of CCL19 to suppress angiogenesis by downregulating VEGF-A expression in CRC cells (53). So, numerous studies have provided us with data upon which to base an investigation of the role of CXCL7 in angiogenesis in CRC patients. We chose VEGF as a marker of angiogenesis and used IHC to evaluate its expression in CRC cells. Then, the relationship between CXCL7 and VEGF expression was analyzed, and we found that the two were positively correlated. Based on these results, we conclude that CXCL7 prominently correlates with VEGF and clinicopathological features in CRC patients. Furthermore, univariate analysis showed that high expression of CXCL7 with VEGF is a risk factor that can be considered in prognosis of CRC patients. However, VEGF was not an independent risk factor in CRC patients. The nomogram and its corresponding evaluation index also showed that CXCL7 was associated with outcome in CRC patients. Based on the above results, we suggest that CXCL7 may regulate expression of VEGF in CRC tissues, maybe for the reason that angiogenic functions can be induced by chemokine-regulated inflammatory stimuli (48, 54–56). Because the treatment methods are also very important for the patient’s prognosis, the related surgery, radiotherapy and chemotherapy methods will be taken into consideration for comprehensively exploring the factors which could affect patient survival time in future study. Furthermore, the relationship between CXCL7 and VEGF should be further studied *in vivo* and *in vitro*, including in CRC serum and cell lines.

In summary, this study analyzed the correlation between CXCL7 and VEGF, and illustrated that co-expression of the two markers leads to a poor prognosis in CRC patients. As chemokines have been suggested to be therapeutic targets in many studies (57, 58), CXCL7 may be a new target for regulating angiogenic signaling pathways, especially those controlling VEGF expression.

## Data Availability Statement

The original contributions presented in the study are included in the article/**Supplementary Material**. Further inquiries can be directed to the corresponding authors.

## Author Contributions

LL, KJ, DPL, DXL, GD, ST, GW, and XL conceived the study. LL, DPL, and ST performed the experiments and were responsible for statistical analysis. LL, KJ, and DPL were in charge of writing review, revision and other related work in this study. All authors contributed to the article and approved the submitted version.

## Funding

This study was supported by the Bozhou science and technology major special projects (No. bzzd2020012), the Bozhou Key R&D projects (No.bzzc2021020) and the incubation project of Bozhou People’s Hospital (No.Byf202017).

## Conflict of Interest

The authors declare that the research was conducted in the absence of any commercial or financial relationships that could be construed as a potential conflict of interest.

## Publisher’s Note

All claims expressed in this article are solely those of the authors and do not necessarily represent those of their affiliated organizations, or those of the publisher, the editors and the reviewers. Any product that may be evaluated in this article, or claim that may be made by its manufacturer, is not guaranteed or endorsed by the publisher.

## Supplementary Material

Supplementary Figure 1 Flowchart for inclusion and exclusion of CRC patients.

## Abbreviations

CRC, colorectal cancer; VEGF, vascular endothelial growth factor; IHC, immunohistochemical; TNM, tumor-node-metastasis; T, depth of tumor invasion; N, nodal involvement; M, distant metastasis; OS, overall survival time; C-index, concordance index; ROC, receiver operating characteristic; Mean (M) and standard deviation (SD); AUC, area under the ROC curve; HR, high risk factor.
