# Supplementary figures and images for: The Chemokine CXCL7 Is Related to Angiogenesis and Associated With Poor Prognosis in Colorectal Cancer Patients
Source: Front Oncol. 2021 Oct 8;11:754221. doi: 10.3389/fonc.2021.754221 (PMC8531515; doi:10.3389/fonc.2021.754221)

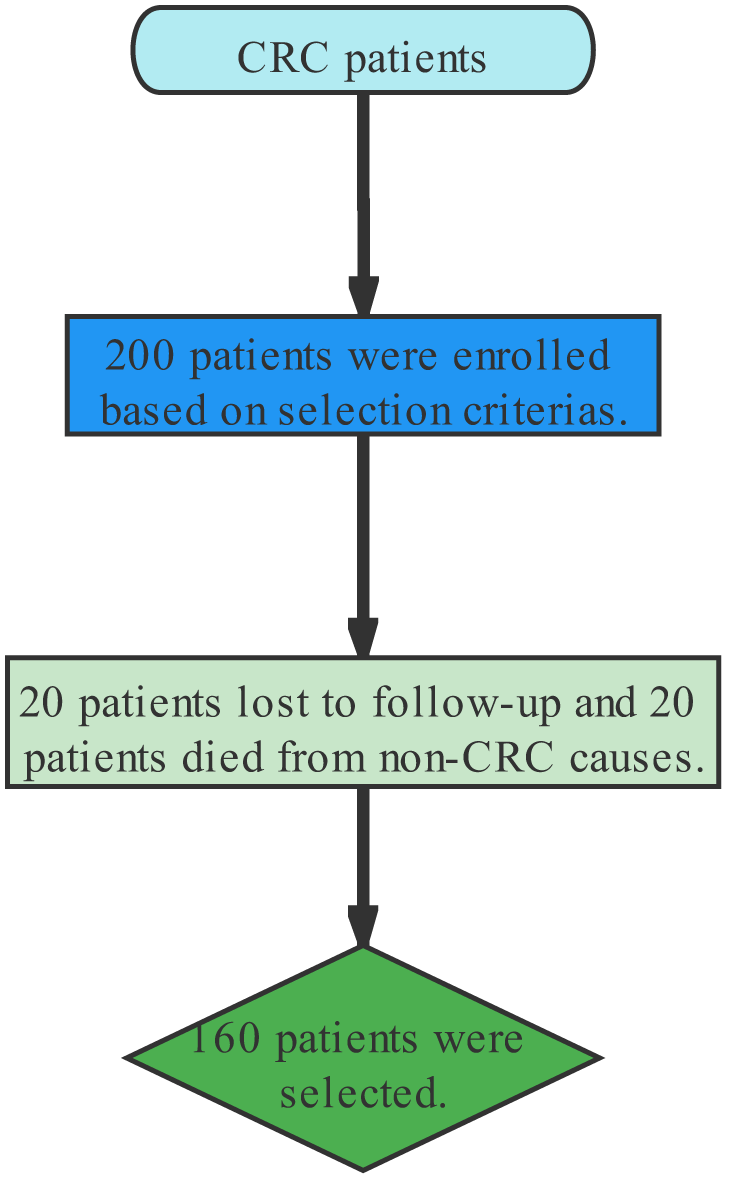

Supplement: Supplementary Figure 1 — Flowchart for inclusion and exclusion of CRC patients. [file Image_1.tif]
